# Supplementary material for: Zymosan-Induced Murine Peritonitis Is Associated with an Increased Sphingolipid Synthesis without Changing the Long to Very Long Chain Ceramide Ratio
Source: Int J Mol Sci. 2023 Feb 1;24(3):2773. doi: 10.3390/ijms24032773 (PMC9917615; doi:10.3390/ijms24032773)
Supplement: Supplementary file 1 [file ijms-24-02773-s001.zip › Supplementary_Figure.pdf]

Figure S1

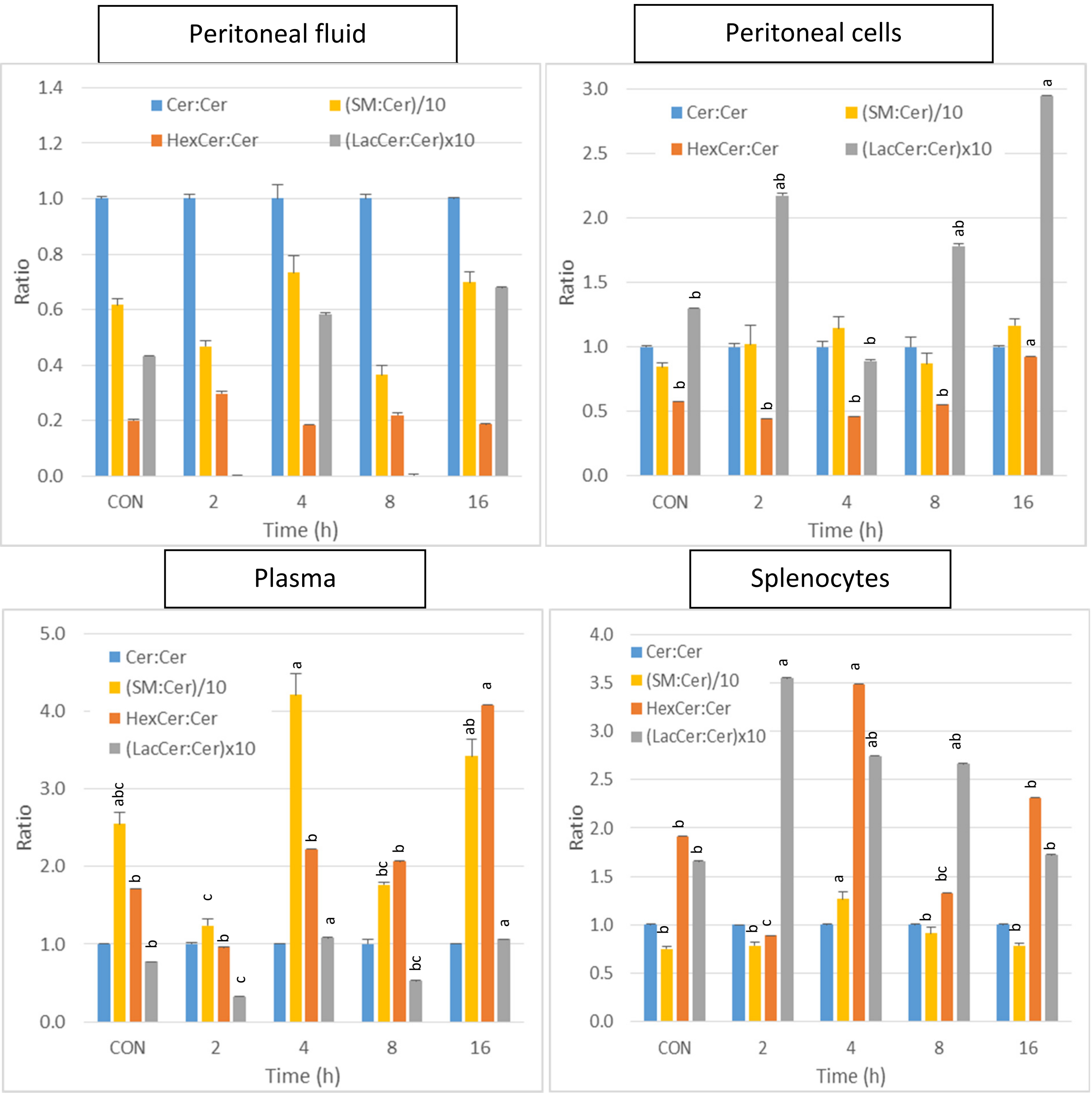


**Figure S1.** Sphingolipid ratios measured in control mice that received PBS (CON) and in mice after intraperitoneal administration of zymosan at a dose of 0.1 mg/mouse at 2, 4, 8, and 16 h post-zymosan injection. Ceramides to ceramides (Cer:Cer), sphingomyelins to ceramides (SM:Cer), hexosylceramides to ceramides (Hex:Cer), and lactosylceramides to ceramides (LacCer:Cer) ratios measured in the peritoneal fluid, peritoneal cells, plasma and the spleen. SM:Cer and LacCer:Cer ratios were corrected by a factor of 10 to take into account the different abundance in SM and LacCer compared to Cer, and permits a better comparison. Values are reported as means ± SE, n = 16 for the controls and n = 4, 4, 4, and 8 for the mice treated at 2, 4, 8, and 16 h post-zymosan injection, respectively. Significant differences among groups were investigated using one way ANOVA. Statistically different groups (Duncan) were then identified using different apex letters ($p<0.05$). Any two means having a common letter, are not significantly different at the 5% level of significance.
